# Gigantic mammary Paget’s disease of a very elderly woman

**DOI:** 10.1186/s40792-018-0541-1

**Published:** 2018-11-16

**Authors:** Kenichi Shibata, Shintaro Nozu, Takayuki Tanaka, Wataru Kimura

**Affiliations:** Department of Gastroenterological, General, Breast and Thyroid Surgery, Yamagata University Faculty of Medicine, 2-2-2, Iida-Nishi, Yamagata-city, Yamagata, 990-9585 Japan

**Keywords:** Breast cancer, Mammary Paget’s disease, Elderly patient

## Abstract

**Background:**

Recently, surgical stress due to breast cancer has been reduced, more so for elderly patients. However, an expanded resection is still required in some situations.

**Case presentation:**

We present a case of a 90-year-old woman with a 15 × 15 cm^2^ erythema and localized skin ulceration with hemorrhage in her right breast. A punch biopsy indicated mammary Paget’s disease. Computed tomography showed that the tumor was only located on the surface of the breast with no metastasis, including of the axillar lymph nodes. We decided to perform surgery with sufficient informed consent. We performed muscle-sparing mastectomy with sampling of an axillar lymph node, adding two stress-relaxation sutures to avoid diastasis. The patient’s postoperative course was good. A histological examination revealed mammary Paget’s disease without invasion and no evidence of a residual tumor of the entire stumps. Her quality of life was improved after surgery.

**Conclusion:**

Mammary Paget’s disease has a relatively good prognosis. However, advanced mammary Paget’s disease leads to a decrease of quality of life with symptoms such as skin ulcer and bleeding. Surgery should be performed in such cases, considering the risks and benefits, even in older patients with comorbidities.

## Background

Mammary Paget’s disease was first described by Sir James Paget in 1876 [1]. Mammary Paget’s disease is a relatively uncommon form of breast cancer that is characterized by an eczematous eruption and ulceration of the nipple that affects the areola; Mammary Paget’s disease estimated to be present in 1–2% of all breast cancers [2, 3]. Its frequency in elderly woman over 80 is approximately 13.9% of all mammary Paget’s disease [3]. Those symptoms worsen patients’ quality of life. But cases of large disease over areola with bleeding are rarely reported and frequency of it is unclear. Since an underlying intraductal spread or invasive carcinoma is frequently detected by a histopathological examination, mastectomy had been considered to be the standard treatment of mammary Paget’s disease [2]. Recently, breast-conserving surgery with sentinel node biopsy has become feasible instead of a conventional mastectomy if this disease is diagnosed appropriately and secure treatments are obtained [2–5]. However, even now, mastectomy is still required for effective treating this disease that missed time to early diagnosis. If patient’s quality of life declines due to symptoms associated with cancer such as skin ulcer and bleeding in the breast, appropriate surgical techniques improve quality of life. However, elderly people with comorbidities may find it difficult to successfully select surgical procedures and anesthesia. We report here that we could successfully resected a gigantic mammary Paget’s disease of a very elderly woman.

We report a rare case of a very elderly woman with gigantic mammary Paget’s disease without invasion to underlying tissue who underwent a mastectomy to improve her quality of life.

## Case presentation

**Fig. 1:**
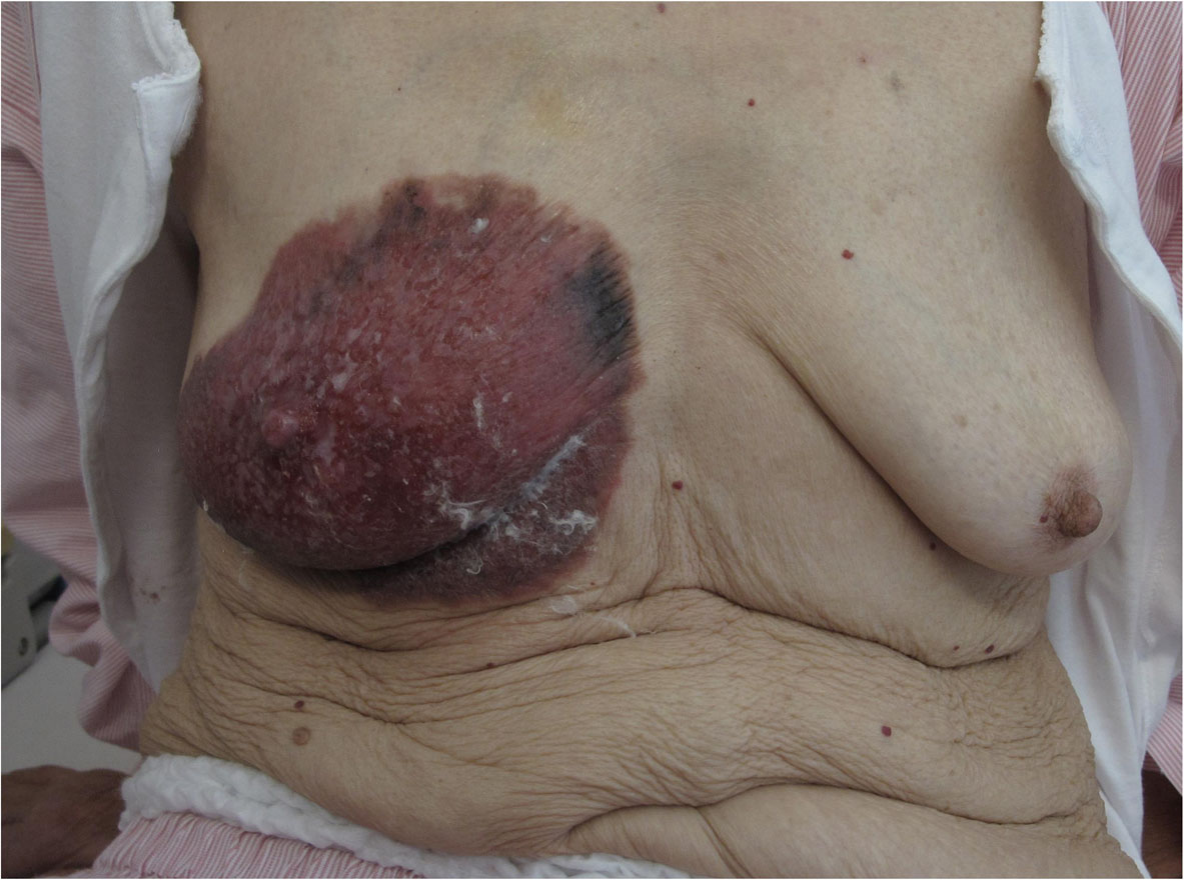
Macroscopic view of the right breast. The area of the tumor was 15 × 15 cm^2^ with erythema and a localized skin ulceration

A 90-year-old woman visited our hospital due to a large area of erythema and localized skin ulceration with hemorrhage of her right breast. Her breast symptoms arose 5 years ago and had been worsening. She could not visit a hospital because she expected for naturally healing and feared noticing cancer and death. She had a history of atrial fibrillation and cerebral infarction 2 months earlier, then her breast lesion was found out. The area of erythema was 15 × 15 cm^2^. Her nipple and alveolar complex were destroyed and had an uncertain shape. Her skin erythema was soft, and no tumor was palpable (Fig. 1). Her quality of life had got worse by hemorrhage and exudate from the tumor, and she felt strong anxiety about getting more worse and death from the cancer.

**Fig. 2:**
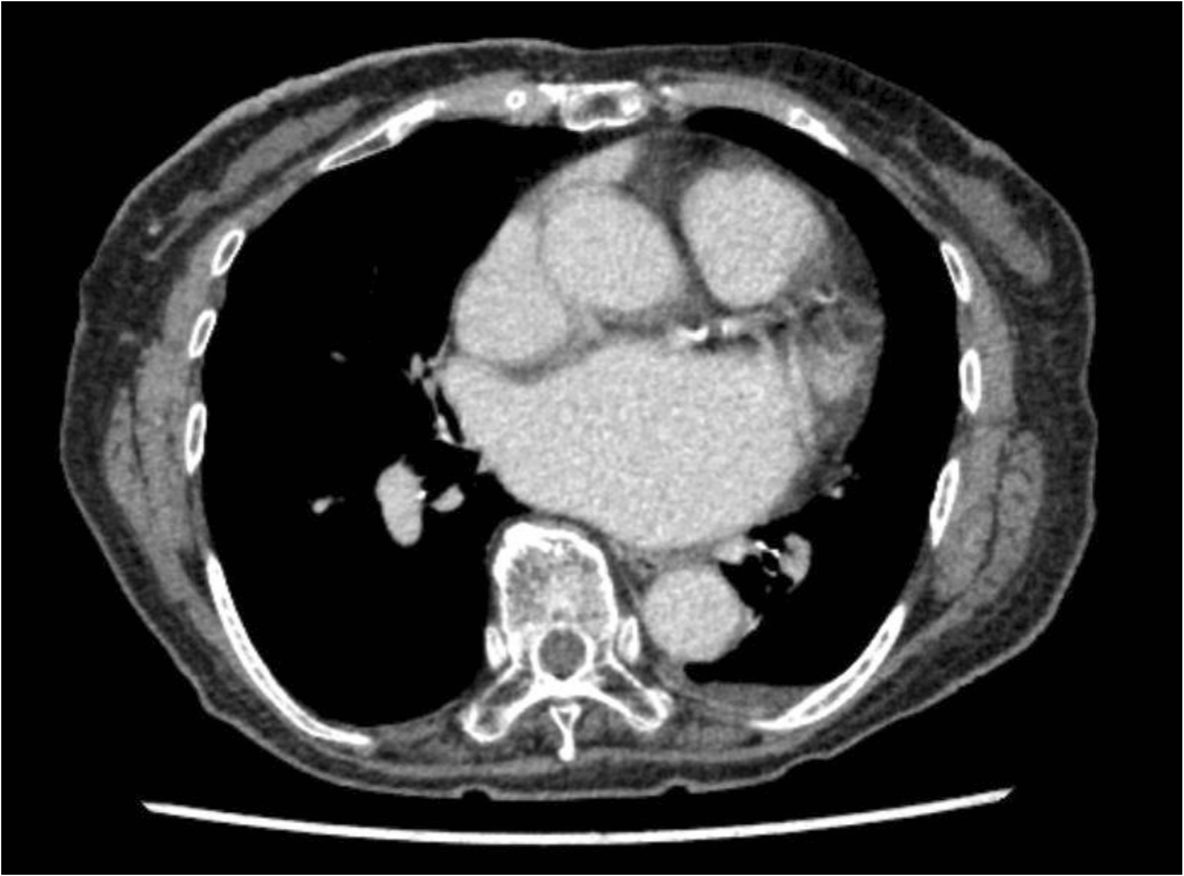
Computed tomography. The tumor was only on the surface of the breast without any underlying tumor

A punch biopsy indicated mammary Paget’s disease. Computed tomography showed that the tumor was only on the surface of the breast, with no metastasis including of the axillar lymph nodes. There was no underlying tumor in the breast (Fig. 2).

**Fig. 3:**
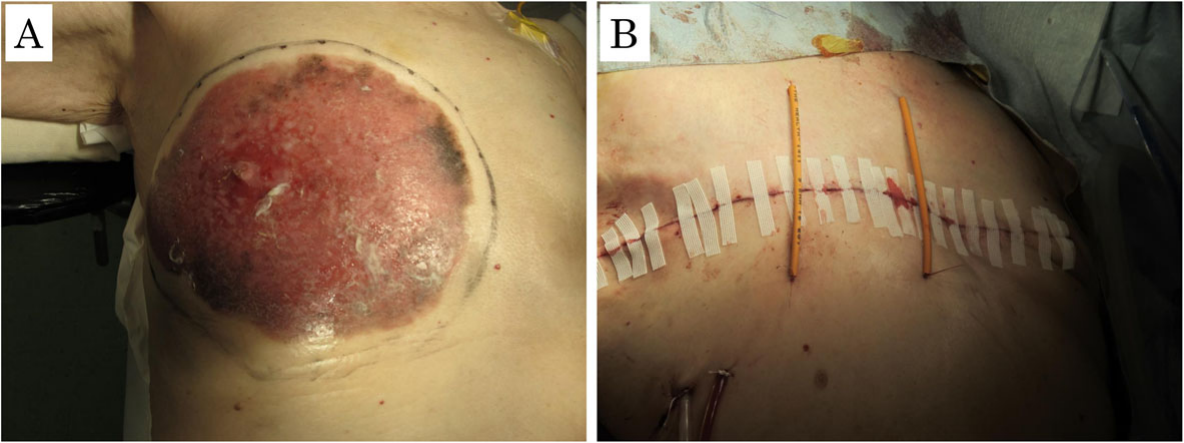
Surgical findings. **a** Muscle-sparing mastectomy with a 1-cm margin from the edge of the skin erythema. **b** Two stress-relaxation sutures to avoid diastasis

Even though our patient was a very elderly woman with comorbidities and her prognosis was relatively good, her symptoms were intolerable. By the request of her and her family, we decided to perform surgery to eliminate the area of erythema after receiving sufficient informed consent. The surgical treatment was performed by two teams that included surgeons and dermatologists. We drew a resection line 1 cm from the skin erythema. Dermatologists were on standby in case a skin graft was needed. We performed muscle-sparing mastectomy with sampling of an axillar lymph node. We added two stress-relaxation sutures to avoid diastasis because the excision area was very large and the tension of the skin flap was strong (Fig. 3a, b). Fortunately, a skin graft was not necessary and her postoperative course was good. The skin flap did not develop major complications such as necrosis, seroma, wound infection, and highly disturbance of moving the right upper limb. We removed the stress-relaxation sutures 7 days after surgery.

**Fig. 4:**
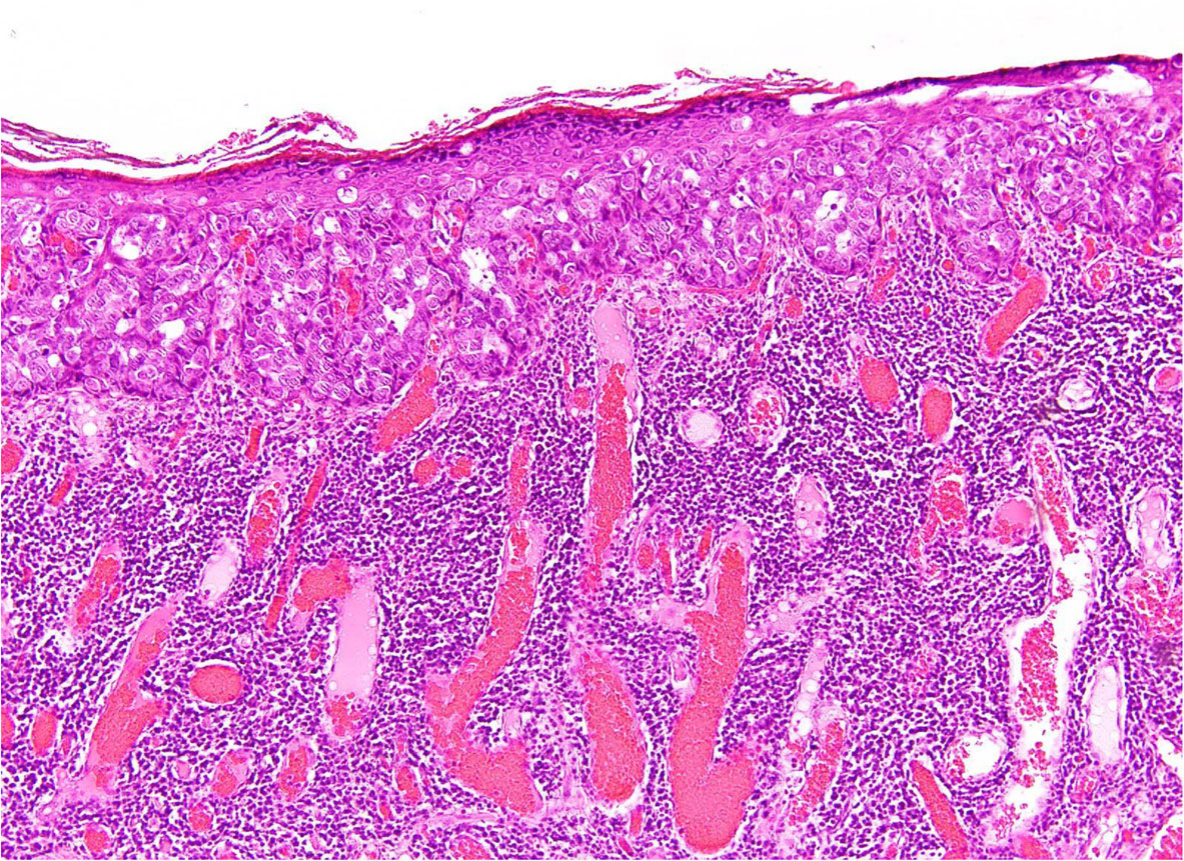
Histological findings of the resected specimen (original magnification, × 10). Characteristic Paget cells were detected within the epidermis. No infiltration was observed

A histological examination revealed mammary Paget’s disease without invasion to underlying tissues (Fig. 4), no evidence of a residual tumor of the entire stumps, and no metastasis in the lymph node. Although she felt a little tightness of the surgical site, paresthesia of the chest wall, and a sense of breast loss, her quality of life improved after surgery by being freed from symptoms and anxiety related to malignancy. It was a great value for her, even if she suffered from these complications.

## Discussion

Mammary Paget’s disease is sometimes difficult to diagnose. Because of its features and the resolution of the eczematous changes with or without application of corticosteroids, mammary Paget’s disease may be mistaken for a benign skin condition, such as dermatitis, delaying the diagnosis [6]. Many case reports indicate that mammary Paget’s disease is sometimes pigmented and mimicking malignant melanoma. But inverse case is rare. Only Lin et.al reported malignant melanoma of the nipple mimicking Paget’s disease, and the correct diagnosis requires histological interpretation [7]. We think that pigmented tumor of the nipple should be firstly suspected mammary Paget’s disease rather than malignant melanoma and should be diagnosed by histopathological examination. However, in our case, it was not difficult to diagnose this disease. Elderly patients sometimes do not go to the hospital in a timely fashion.

Mammary Paget’s disease generally requires a mastectomy, even if it is localized around the nipple and alveolar complex. Recently, breast-conserving mastectomy for treating mammary Paget’s disease has become feasible instead of conventional mastectomy. However, resection of a large area is sometimes necessary. Modern surgical and anesthetic techniques allow breast cancer surgery to be performed safely in nearly all elderly patients. In elderly patients, the majority of the operative risk is due to an underlying comorbidity and the effects of anesthesia on dysfunctional organ systems. Optimization of the anesthetic technique can minimize the operative risk [8, 9]. If the patient’s disease had been localized, resection under local anesthesia would have been performed. However, the area of the tumor was large, so we performed mastectomy under general anesthesia after receiving informed consent. There is a previous report of a large area affected by mammary Paget’s disease. G. Nicoletti et al. reported a 79-year-old patient with a gigantic mammary Paget’s disease with an underlying invasion that required a myocutaneous flap [10]. The patient was relatively young, unlike the patient in our case. We considered a skin graft and myocutaneous flap to be too high risk for our patient. If a myocutaneous flap had been essential, we would not have performed the surgery. Topaz M et al. reported that stress-relaxation sutures can be used for immediate primary closure of large soft tissue defects [11]. The concept of using these sutures was developed a long time ago, but the effective use and progress of this technique are still applicable to the present day. We used two stress-relaxation sutures. However, it might not be necessarily fundamental to this patient, we believe that the sutures were needed to reduce the risk of surgery. As far as we know, the patient in this report is the eldest woman of mammary Paget’s disease treated by surgery despite the absence of invasion to underlying tissues. As the population is aging, cases like this may occur more frequently.

Since her tumor showed neither an underlying invasion nor lymph node metastasis, her prognosis was relatively good and not life-threatening. Her symptoms would persist over a long period of time. Although she was a very elderly woman with comorbidities, we decided to perform surgery to implove her quality of life. Despite her life expectancy being unclear, the surgery we performed was not meaningless, as its aim was to improve her quality of life.

## Conclusion

Mammary Paget’s disease has a relatively good prognosis. However, advanced mammary Paget’s disease leads to a decrease of quality of life with symptoms such as skin ulcer and bleeding. Surgery should be performed in such cases considering the risks and benefits, even in older patients with comorbidities.
